# Medium-long term prognosis prediction for idiopathic pulmonary fibrosis patients based on quantitative analysis of fibrotic lung volume

**DOI:** 10.1186/s12931-022-02276-3

**Published:** 2022-12-22

**Authors:** Kaifeng Du, Yichun Zhu, Ruolin Mao, Yubei Qu, Bo Cui, Yuan Ma, Xin Zhang, Zhihong Chen

**Affiliations:** 1 Department of Respiratory and Critical Care Medicine, Zhongshan Hospital, Fudan University, Shanghai Institute of Respiratory Disease, Shanghai, 200032 China; 2 Department of Respiratory and Critical Care Medicine, Minhang Hospital, Fudan University, Shanghai, 201199 China

**Keywords:** Idiopathic pulmonary fibrosis, Prognosis prediction, Quantitative analysis, Pulmonary function test

## Abstract

**Purpose:**

To investigate the prognostic value of quantitative analysis of CT among patients with idiopathic pulmonary fibrosis (IPF) by quantifying the fibrosis extent and to attempt to provide precise medium-long term prognostic predictions for individual patients.

**Methods:**

This was a retrospective cohort study that included 95 IPF patients in Zhongshan Hospital, Fudan University. 64 patients firstly diagnosed with IPF from 2009 to 2015 was included as the derivation cohort. Information regarding sex, age, the Gender-Age-Physiology (GAP) index, high-resolution computed tomography (HRCT) images, survival status, and pulmonary function parameters including forced vital capacity (FVC), FVC percent predicted (FVC%pred), diffusing capacity of carbon monoxide (DLCO), DLCO percent predicted (DLCO%pred), carbon monoxide transfer coefficient (KCO), KCO percent predicted (KCO%pred) were collected. 31 patients were included in the validation cohort. The Synapse 3D software was used to quantify the fibrotic lung volume (FLV) and total lung volume (TLV). The ratio of FLV to TLV was calculated and labeled CT_FLV/TLV%_, reflecting the extent of fibrosis. All the physiological variants and CT_FLV/TLV%_ were analyzed for the dimension of survival through both univariate analysis and multivariate analysis. Formulas for predicting the probability of death based on the baseline CT_FLV/TLV%_ were calculated by logistic regression, and validated by the validation cohort.

**Results:**

The univariate analysis indicated that CT_FLV/TLV%_ along with DLCO%pred, KCO%pred and GAP index were significantly correlated with survival. However, only CT_FLV/TLV%_ was meaningful in the multivariate analysis for prognostic prediction (HR 1.114, 95% CI 1.047–1.184, P = 0.0006), and the best cutoff was 11%, based on receiver operating characteristic (ROC) curve analysis. The survival times for the CT_FLV/TLV%_ ≤ 11% and CT_FLV/TLV%_ > 11% groups were significantly different. Given the CT_FLV/TLV%_ data, the death probability of a patient at 1 year, 3 years and 5 years could be calculated by using a particular formula. The formulas were tested by the validation cohort, showed high sensitivity (88.2%), specificity (92.8%) and accuracy (90.3%).

**Conclusion:**

Quantitative volume analysis of CT might be useful for evaluating the extent of fibrosis in the lung. The CT_FLV/TLV%_ could be a valuable biomarker for precisely predicting the medium-long term prognosis of individual patients with IPF.

## Introduction

Idiopathic pulmonary fibrosis (IPF) is the most common type of idiopathic interstitial pneumonia and is defined as a spontaneously occurring (idiopathic) specific form of chronic fibrosing interstitial pneumonia limited to the lung according to an American Thoracic Society/European Respiratory Society (ATS/ERS) consensus statement [1]. IPF has a usual interstitial pneumonia (UIP) pattern on high-resolution computed tomography (HRCT) images or surgical lung biopsy. IPF is a deadly disease with poor prognosis. The median survival of IPF has been reported to range from 2 to 5 years [2, 3]. However, the prognosis varies among individual patients with IPF. The risk of death in individual IPF patients at the time of diagnosis ranges from < 1 year to > 10 years [2]. Accurate prediction of survival is critical for guiding clinical care.

The known predictors of reduced survival include older age, male sex, lower forced vital capacity (FVC) percent predicted (FVC%pred), lower diffusing capacity of carbon monoxide (DLCO) percent predicted (DLCO%pred), need for supplemental oxygen, greater severity of dyspnea, lower distance walked on the six-minute walk test (6MWT), more respiratory hospitalization and greater extent of fibrosis on HRCT images [3–5]. Several index models, including the Gender-Age-Physiology (GAP) index [3], the composite physiologic index (CPI) [6] and a risk stratification score (ROSE) [5], have been proposed. All these models include variables of FVC and DLCO. However, achieving consistent DLCO results between and within laboratories remains a difficult problem; even when tested in the same laboratory a few days apart, DLCO results from healthy subjects may vary as much as 8 mL/min per mmHg [7]. Furthermore, qualified pulmonary function tests (PFTs) are sometimes unavailable for elderly patients or those who cannot cooperate. HRCT is much easier to perform for these patients and more accurate. In previous studies, it was confirmed that the CT fibrosis score by visual assessment or computer-based algorithms could replace standard clinical and physiological variables [8–14]. Quantifying the severity of fibrosis with HRCT is a promising approach for predicting the prognosis of patients with IPF, especially the computer-based assessment, with more efficiency and accuracy as compared with artificial visual assessment.

In our study, we focused on the value of quantitative volume analysis of CT in the quantification of fibrosis, calculated the fibrotic lung volume (FLV), total lung volume (TLV) and FLV/TLV ratio (CT_FLV/TLV%_), and then used the CT_FLV/TLV%_ to predict the precise death probability within 5 years for individual patients with IPF.

## Methods

### Study population

This was a retrospective cohort study of patients diagnosed with stable IPF at Zhongshan Hospital, Fudan University. Two cohorts were collected, including a derivation cohort firstly diagnosed as IPF between 1 January 2009 and 31 December 2015, as well as a validation cohort firstly diagnosed as IPF between 1 January 2009 and 31 December 2020. According to the diagnosis criteria from ATS/ERS/JRS/ALAT 2011, the inclusion criteria of the study included (1) definite features of UIP on HRCT images or (2) surgical lung biopsy results correlated with the HRCT findings. The exclusion criteria were as follows: (1) interstitial lung diseases of known cause, e.g., drug-induced, environmental, occupational or connective tissue diseases; (2) IPF combined with pulmonary infections and needed for anti-infective therapy; (3) other severe systemic diseases or organ dysfunction; or (4) malignant tumors.

The study was approved by the institutional ethics committees of Zhongshan Hospital, Fudan University (ethical number: ZS2013-31). The requirement for informed consent from the patients included in this study was waived due to the retrospective nature of the study, and any personal information from the data was removed beforehand.

### Data collection

General information for the enrolled patients, including sex, age, date of diagnosis, symptoms and complications, as well as lung HRCT images and PFT were collected. PFT parameters included FVC, FVC%pred, DLCO, DLCO%pred, carbon monoxide transfer coefficient (KCO), KCO percent predicted (KCO%pred). The GAP index [3] was calculated based on the sex, age and PFT data.

The patients in the derivation cohort were followed up every 6 months or 1 year either face-to-face or by telephone. Patient enrollment started on 1 January 2009 and ended on 31 December 2015. The whole cohort was followed up until 31 January 2021. The longest observation time was 145 months (12 years and 1 month). This process ensured that the last participant’s observation time was greater than 5 years. For each individual, the observation was ended if the patient died; otherwise, the patient was observed until the end of the study. In the validation cohort, patients firstly diagnosed as IPF between 1 January 2009 and 31 December 2020 were searched from the Hospital Information System. The patients who had already been included in the derivation cohort were excluded from the validation cohort. The survival status was collected by telephone interview between 1 September 2022 to 31 October 2022. If a patient in the validation cohort is diagnosed with IPF for less than 5 years and survives, the patient will be excluded. This ensured that patients who survived were observed for more than 5 years.

### HRCT and quantitative analysis of fibrotic lung volume

**Fig. 1:**
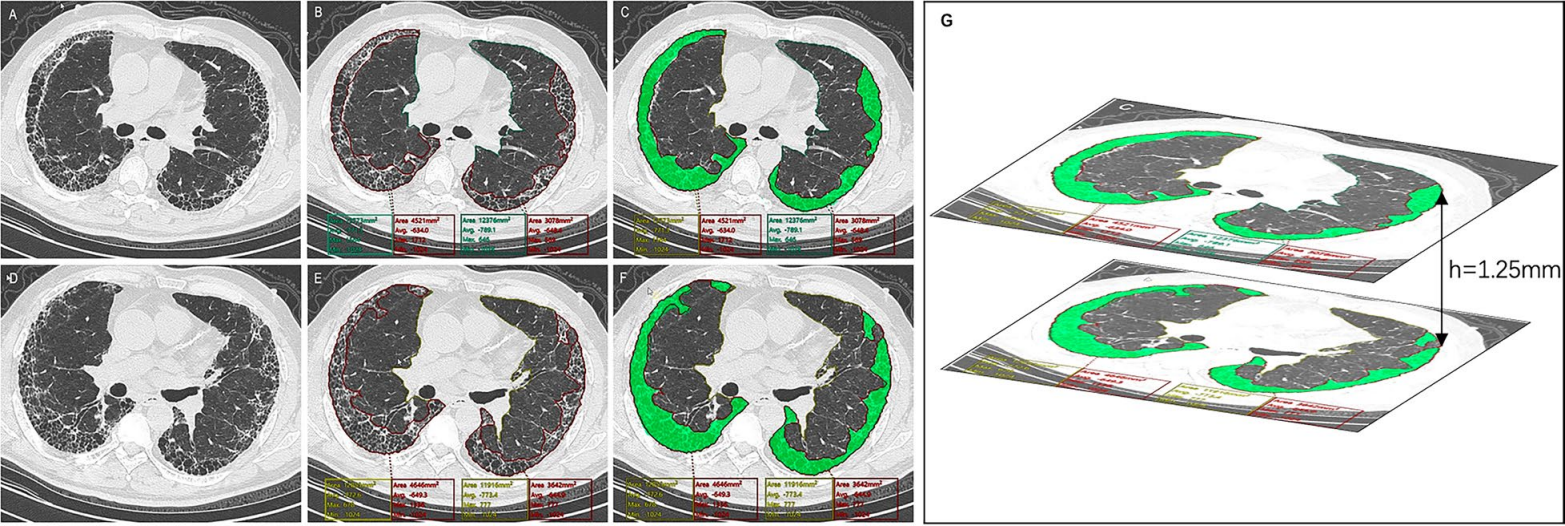
Evaluation of the CT_FLV/TLV%_ by Synapse 3D software. **A** and **D** Two adjacent HRCT images of a transverse axial scan of one IPF patient. **B** and **E** The total lung area and the total area of fibrosis were automatically captured and drawn by the Synapse 3D software. **C** and **F** Green shows the area of fibrosis after drawing by the Synapse 3D software. **G** The FLV and TLV were approximately calculated by the Cylinder volume formula

HRCT imaging was performed using a 64-detector row spiral CT machine (Lightspeed VCT, Ge Healthcare, Hamilton, USA) in the supine position at full inspiration breath hold. The diagnostic settings were as follows: tube voltage, 120 ~ 140 kV; tube current, 140 mAs; collimation, 64 × 0.625 mm; pitch, 0.9875; and reconstruction slice thickness, 1.25 mm. The original images in DICOM format were loaded into image analysis software (Synapse 3D, V4.4, Fuji Film, Japan). Total lung tissue and fibrotic lung tissue were manually delineated layer by layer. Fibrotic lung tissue was defined as honeycombing or reticular opacities on CT and was judged jointly by a respiratory physician (with no less than 10 years of professional experience in respiratory medicine) and a radiologist (with no less than 7 years of professional experience in chest radiology) who did not know any of the patients’ clinical information. If the raters disagreed, a third senior radiologist joined the discussion. The software automatically calculated the total lung tissue area and the fibrotic lung tissue area of each CT layer. According to the approximate cylindrical volume calculation formula, the volume of each lesion and total CT lung volume were computed automatically by the computer-aided diagnosis (CADx) system (Fig. 1). The FLV and TLV were expressed as absolute values, and the FLV/TLV ratio refers to the percentage of FLV relative to TLV (CT_FLV/TLV%_).

### Statistical analysis

Baseline characteristics of the patient cohort were summarized according to survival status. All data are presented as the mean ± SD for continuous variables and as absolute numbers and percentages for categorical data. The differences between two groups were assessed by Student’s t-test or the Mann–Whitney U test for continuous data and the chi-square or Fisher’s exact test for categorical variables.

The correlation between the clinical parameters and survival time was analyzed by linear correlation analysis and was expressed by the Pearson correlation coefficient. The closer the area under the curve (AUC) value is to one, the better the discrimination capacity of the model. Youden's index was used to identify the optimal cutoff value for the CT_FLV/TLV%_, which was 11%. Kaplan–Meier survival curves were calculated in strata defined by the CT_FLV/TLV%_ categories (CT_FLV/TLV%_ ≤ 11%, > 11%). Two-sided log-rank tests defined significance.

To explore risk factors that could be associated with all-cause mortality, a univariate Cox proportional hazards model was initially applied using demographics, clinical variables, physiological indices and CT indices. Then, a multivariate binary logistic regression was performed by introducing the variables selected in the univariate regression model, and the statistically significant variables were eventually identified. Based on the results of the multivariate analysis, mortality predictive formulas were further constructed, and were validated in the validation cohort. The sensitivity, specificity and accuracy of the formulas were calculated.

All tests of hypotheses were two-tailed and conducted at a significance level of 0.05. Statistical analyses were conducted using SAS version 9.3.

## Results

### Patient characteristics

Eighty-one patients with IPF were included in the derivation cohort. Among them, 6 were excluded for malignant tumors (1 with nasopharyngeal cancer and 5 with lung cancer), 2 were excluded for connective tissue disease, and 9 were lost to follow-up. Therefore, 64 patients were included in the analyses of derivation cohort. The number of survivors or deaths was recorded at the timepoint of at least 5 years of observation since enrollment as well as at the end of the study. Forty-five patients completed the baseline PFT, while 19 failed (10 patients lost their initial PFT reports, 9 patients could not complete the PFT process). Among the 45 patients, 37 finished the diffusion function test, and 8 could not cooperate. All 64 patients completed baseline HRCT scans, and 21 patients were followed up with HRCT after 1 and 3 years.

Fifty-six patients were included in the validation cohort. Among them, 1 was excluded for liver cancer, 9 survivors were excluded because the time to first diagnosis of IPF was less than 5 years, and 15 were excluded since loss of communication by telephone. Therefore, 31 patients were finally included in the analyses of validation cohort. Baseline HRCT images were available in all patients, but the PFT reports were available in only 16 patients.

### Correlation of patient characteristics and survival time

The patients in both derivation and validation cohorts were divided into two groups according to their 5-year survival. Table 1 shows the patient characteristics of the cohorts. In the derivation cohort, among 64 patients, 31 (48.4%) died, and 33 (51.6%) survived after 5 years of observation since enrollment. DLCO%pred (39.3 ± 14.4% vs. 57.6 ± 22.4%, P = 0.0033) and KCO%pred (57.6 ± 22.4% vs. 75 ± 21.9%, P = 0.0067) were significantly decreased in the death group compared with the survival group. The mean GAP index was higher in the death group (4.9 ± 1.5 vs. 3.8 ± 2, P = 0.0420). The baseline CT_FLV/TLV%_ showed a significant difference between the two groups: 29.6% ± 11.6% in the death group and 10.7% ± 7.1% in the survival group (P < 0.0001). There were no significant differences between the two groups with regard to sex, age, FVC (absolute value), FVC%pred, DLCO (absolute value) or KCO (absolute value). In the validation cohort, only CT_FLV/TLV%_ showed a significant difference between 5-year survival and death groups (P < 0.0001).

**Table 1:** Patient characteristics and 5-year survival in derivation cohort and validation cohort

Characteristics	Derivation cohort				Validation cohort				P-values between cohorts
	Derivation cohort (n = 64)	5-year survival (n = 31)	5-year death (n = 33)	P-values	Validation cohort (n = 31)	5-year survival (n = 14)	5-year death (n = 17)	P-values	
5-Year mortality	33 (51.6%)				17 (54.8%)				0.8285
Male sex (%)	50 (78.1%)	21 (66.7%)	29 (87.9%)	0.0713	21 (67.7%)	8 (57.1%)	13 (76.5%)	0.4414	0.3181
Age(yr)	69.7 ± 6.7	69.1 ± 6.5	70.3 ± 6.8	0.4847	71.4 ± 8.4	70.4 ± 8.5	72.3 ± 8.5	0.5340	0.2843
FVC(L)	2.3 ± 0.6	2.3 ± 0.7	2.3 ± 0.6	0.8179	2.3 ± 0.8	2.1 ± 0.4	2.4 ± 1.0	0.4290	0.7059
FVC%pred(%)	68.1 ± 16.8	70.7 ± 16.4	65.1 ± 17.2	0.2695	67.3 ± 17.2	63.3 ± 13.8	70.5 ± 19.7	0.4297	0.8708
DLCO(mmol/min/kPa)	5.0 ± 4.2	5.6 ± 4.3	4.3 ± 4.1	0.3286	5.8 ± 4.2	6.3 ± 4.6	4.6 ± 5.5	0.7767	0.5488
DLCO%pred(%)	49.1 ± 21	57.6 ± 22.4	39.3 ± 14.4	0.0033	45.7 ± 17.8	48.5 ± 23.5	43.2 ± 12.7	0.6189	0.5935
KCO(mmol/min/kPa/L)	1.3 ± 1.1	1.5 ± 1.2	1.2 ± 0.9	0.3262	1.5 ± 1.2	1.6 ± 1.7	1.4 ± 0.8	0.7911	0.6226
KCO%pred(%)	66.4 ± 21.7	75 ± 21.9	57.6 ± 22.4	0.0067	57.4 ± 27.3	52.9 ± 37.7	61.9 ± 12.1	0.5595	0.2236
GAP index	4.3 ± 1.8	3.8 ± 2	4.9 ± 1.5	0.0420	4.4 ± 1.8	5.0 ± 1.9	3.9 ± 1.6	0.2459	0.8347
Baseline CT_FLV//TLV%_(%)	20.4 ± 13.5	10.7 ± 7.1	29.6 ± 11.6	< 0.0001	24.5 ± 17.6	10.6 ± 7.2	36.0 ± 15.1	< 0.0001	0.2137

In the derivation cohort, the patients were then divided into three groups according to their survival time (< 2 years, 2–5 years and ≥ 5 years), and the patient characteristics, including sex, age, baseline FVC%pred, DLCO%pred, KCO%pred, GAP index and baseline CT_FLV/TLV%_, were compared among the groups (Table 2). A higher baseline DLCO%pred (P = 0.0048) and KCO%pred (P = 0.0224), a lower GAP index (P = 0.0066), and a lower baseline CT_FLV/TLV%_ (P < 0.0001) were protective factors for patients with IPF when considering the survival time.

**Table 2:** Patient characteristics and survival time in the derivation cohort

Characteristics	Survival time			P-values
	< 2 years	2–5 years	≥ 5 years	
Number	22	21	21	
Male sex(%)	18(81.82%)	18(85.71%)	14(66.67%)	0.3559
Age(yr)	70.7 ± 6.8	69.4 ± 5.9	69 ± 7.4	0.6848
FVC(L)	2.3 ± 0.7	2.3 ± 0.6	2.4 ± 0.7	0.7449
FVC%pred(%)	63.2 ± 19.6	65.9 ± 15.2	74.4 ± 15.1	0.168
DLCO%pred(%)	40.1 ± 17.7	41.5 ± 13	61.9 ± 23.3	0.0048
DLCO(mmol/min/kPa)	4.1 ± 4.8	4.5 ± 3.4	6.1 ± 4.6	0.4047
KCO(mmol/min/kPa/L)	1.1 ± 1.1	1.3 ± 0.9	1.6 ± 1.2	0.5353
KCO%pred(%)	56.7 ± 24.7	60.2 ± 15	78.6 ± 21.8	0.0224
GAP index	5.1 ± 1.7	4.8 ± 1.4	3.2 ± 1.9	0.0066
Baseline CT_FLV/TLV%_(%)	32.2 ± 11.6	20.8 ± 10.2	7.7 ± 3	< 0.0001

**Fig. 2:**
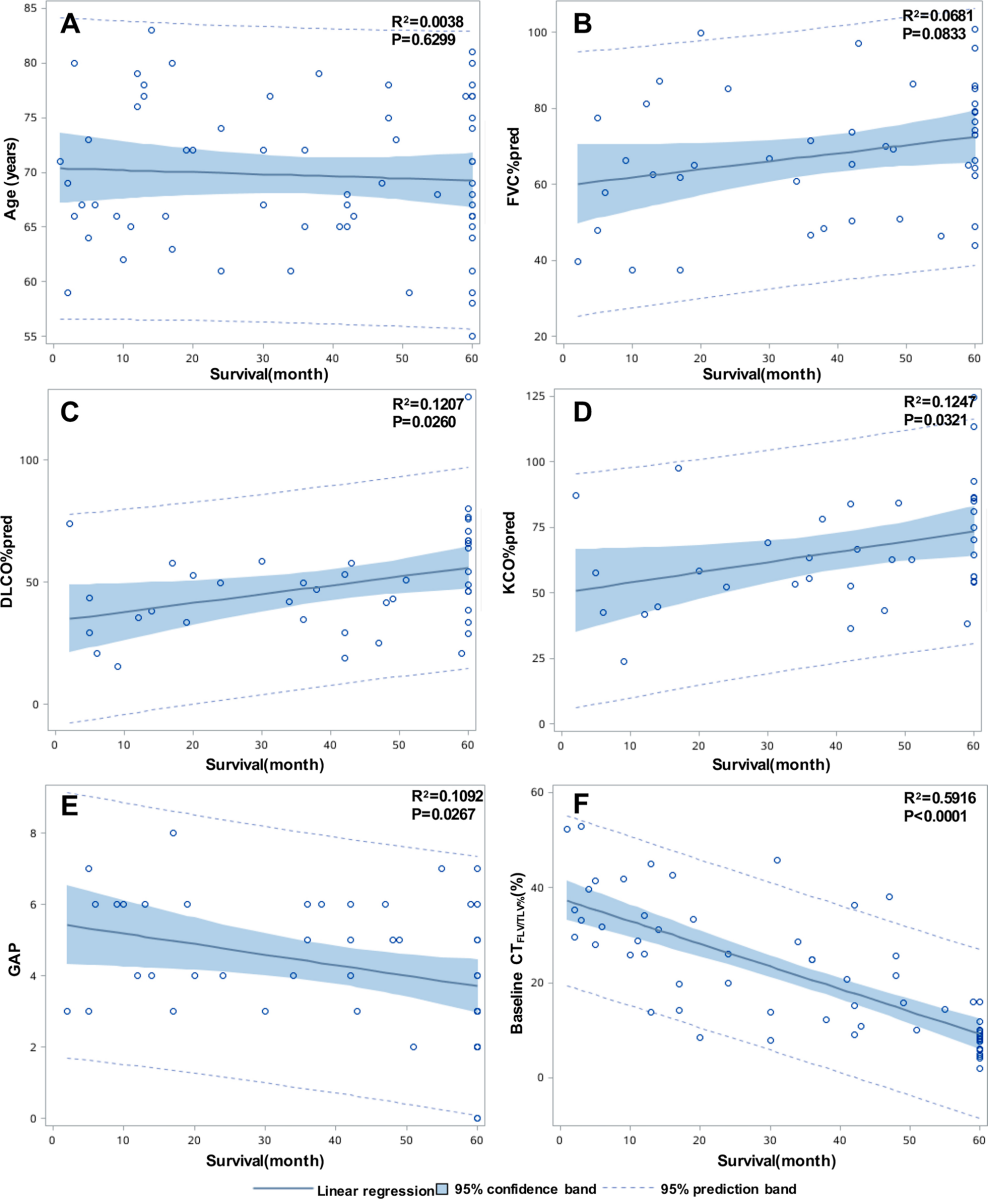
Correlation between the survival time and patient characteristics. Graphs show that age (**A**) and FVC%pred (**B**) do not correlate with the survival time. DLCO%pred (r^2^ = 0.1207, P = 0.0260) (**C**) and KCO%pred (r^2^ = 0.1247, P = 0.0321) (**D**) positively correlated with survival. The GAP index (**E**) negatively correlated with survival (r^2^ = 0.1092, P = 0.0267). The baseline CT_FLV/TLV%_ (**F**) showed a strongly negative correlation with survival (r^2^ = 0.5916, P =  < 0.0001)

The linear correlation analysis of survival time and patient characteristics showed similar results (Fig. 2). DLCO%pred (r^2^ = 0.1207, P = 0.0260) and KCO%pred (r^2^ = 0.1247, P = 0.0321) were positively correlated with survival time, while the GAP index had a negative correlation (r^2^ = 0.1092, P = 0.0267). The baseline CT_FLV/TLV%_ had a strong negative correlation with the survival time (r^2^ = 0.5916, P < 0.0001). Age and FVC%pred showed no significant correlation with the survival time.

Therefore, we suggest that the baseline DLCO%pred, KCO%pred, and GAP index, and especially the baseline CT_FLV/TLV%_, are predictors of the survival time for patients with IPF.

### Prediction of mortality probability by CT_FLV__/TLV%_

**Fig. 3:**
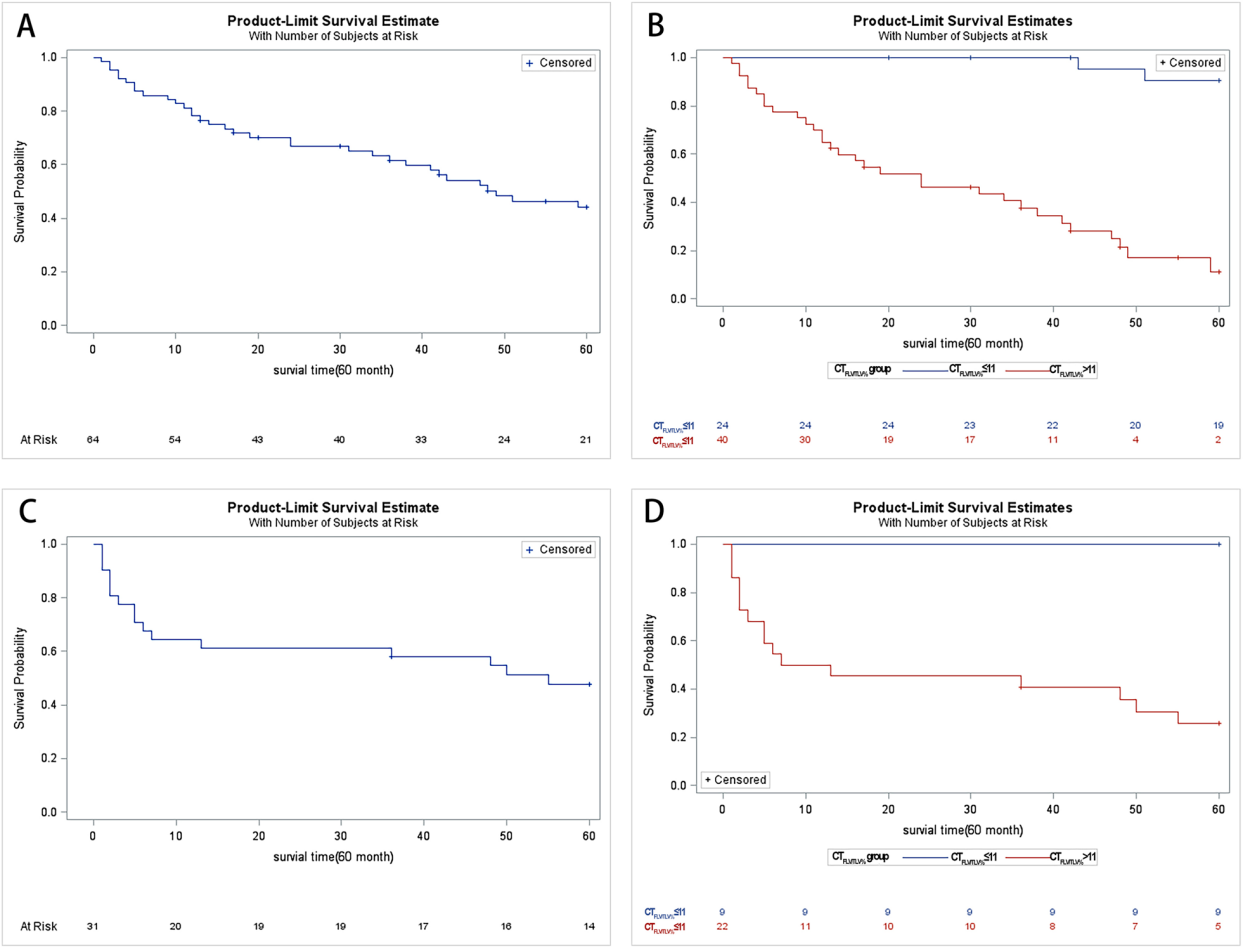
Kaplan–Meier analysis of the survival of patients. **A** The Kaplan–Meier curve of 64 patients in the derivation cohort. **B** The 64 patients in the derivation cohort were separated into two groups according to the baseline CT_FLV/TLV%_, with a cutoff of 11%. The two curves separate obviously at the beginning of the follow-up and become farther with the increase in follow-up months. **C** The Kaplan–Meier curve of 31patients in the validation cohort**. D** Patients in the validation cohort were divided into two groups by the cutoff point of 11% for baseline CT_FLV/TLV%_. The two survival curves separated apart significantly even at the beginning of the observation. No patient of the CT _FLV/TLV%_ < 11% group died in the first 5 years. The graph only shows the survival in the first 5 years from the date of enrollment of every individual patient in our study

A univariate Cox proportional hazards model was applied in the derivation cohort to explore the risk factors associated with 5-year all-cause mortality. The variants included the baseline age, FVC, FVC%pred, DLCO, DLCO%pred, KCO, KCO%pred, GAP index, and baseline CT_FLV/TLV%_. Among these variants, the DLCO%pred, KCO%pred and CT_FLV/TLV%_ were considered significant variants. Multivariate Cox analysis was further performed, covariates included sex, age, FVC%pred, DLCO%pred, KCO%pred, GAP index, and baseline CT_FLV/TLV%_, and only baseline CT_FLV/TLV%_ was a significant predictor of 5-year mortality (HR 1.114, 95% CI 1.047–1.184, P = 0.0006). The Kaplan–Meier survival curve demonstrated a significant difference in mortality at 5 years, which was defined by a cutoff point of 11% for CT_FLV/TLV%_ (Fig. 3A and B).

In the validation cohort, patients were divided into two groups by the cutoff point of 11% for baseline CT_FLV/TLV%_. The Kaplan–Meier survival curve also showed significant difference between groups (Fig. 3C and D).

**Fig. 4:**
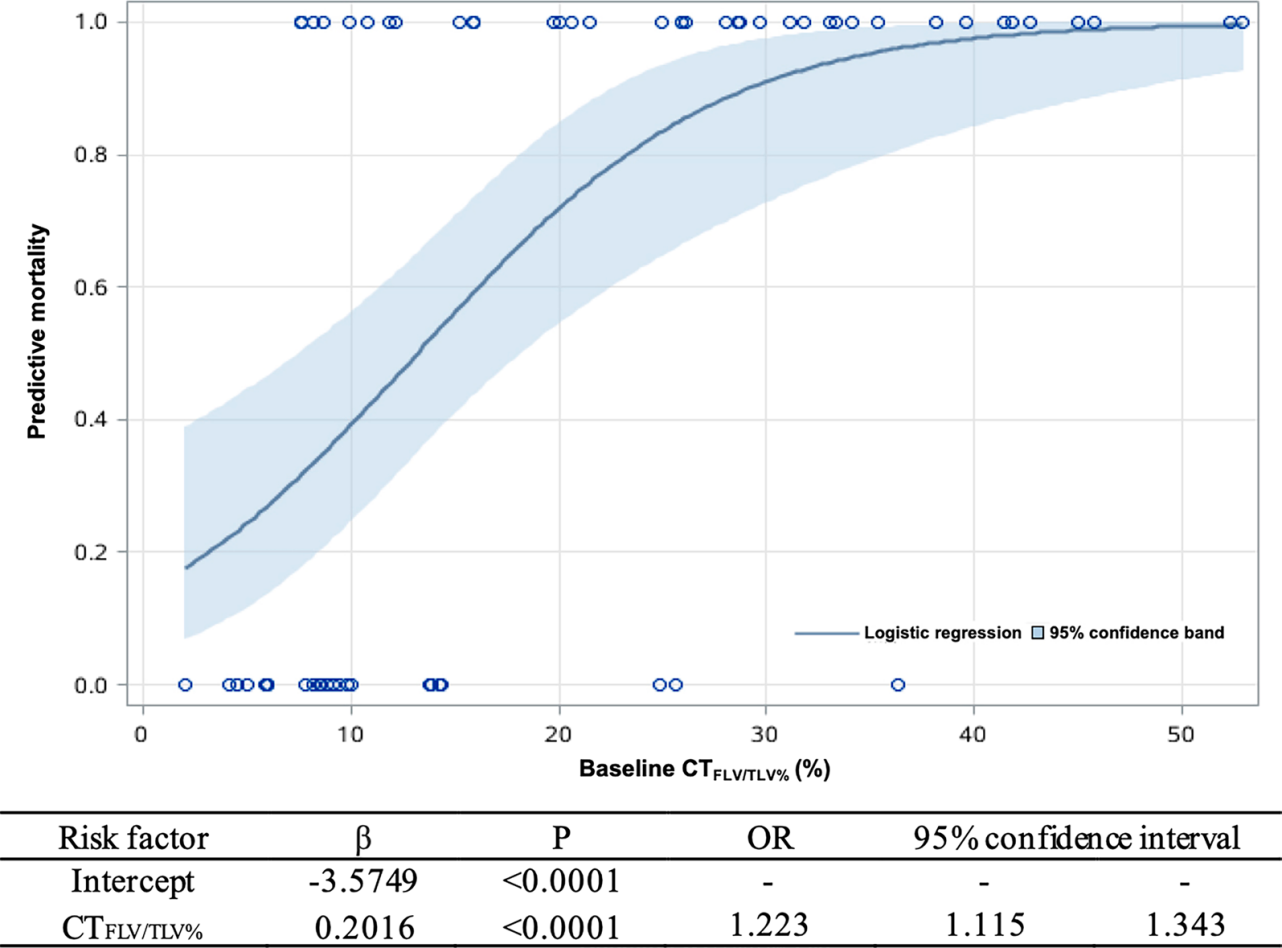
Five-year mortality prediction based on the baseline CT_FLV/TLV%_. A logistic model was applied to create a predictive formula of 5-year mortality. The logistic regression line and the 95% confidence band are shown in the graph

Based on the survival data of the derivation cohort, a logistic model was applied to create a predictive formula of the probability of death. Figure 4 shows the logistic regression curve for the prediction of the 5-year death probability. The logistic equation was as follows:

$$A = \mathop \sum \limits_{i = 0}^{p} \beta_{i} X_{i} = - 3.5749 + 0.2016 \times CT_{{{\text{FLV}}/{\text{TLV\% }}}}$$

$$\hat{P} = \frac{1}{{1 + \exp \left( { - \mathop \sum \nolimits_{i = 0}^{p} \beta_{i} X_{i} } \right)}}$$

where $\hat{P}$ is the 5-year death probability. It is clear from the equation that the risk of death increased directly with the increase in the baseline CT_FLV/TLV%_.

For instance, the baseline CT_FLV/TLV%_ of a patient with IPF was 19.99%, and that patient’s 5-year death probability $\left( {\hat{P}} \right)$ was as follows:

$${\text{A}} = - \,3.5749 + 0.2016 \times 19.99 = 0.455084$$

$$\hat{P} = \frac{1}{{1 + {\text{exp}}\left( { - \,0.455084} \right)}} = 63.4\%$$

Furthermore, we calculated the prediction formula for the 1-year and 3-year death probabilities based on the cohort data for the CT_FLV/TLV%_, 1-year death and 3-year death by logistic regression.

The prediction of the 1-year death probability was as follows:

$$\mathop \sum \limits_{i = 0}^{p} \beta_{i} X_{i} = - \,4.7344 + 0.1342 \times CT_{{{\text{FLV}}/{\text{TLV\% }}}}$$

$$\hat{P} = \frac{1}{{1 + {\text{exp}}\left( { - \mathop \sum \nolimits_{i = 0}^{p} \beta_{i} X_{i} } \right)}}$$

The prediction of the 3-year death probability was as follows:

$$\mathop \sum \limits_{i = 0}^{p} \beta_{i} X_{i} = - \,5.7881 + 0.2376 \times CT_{{{\text{FLV}}/{\text{TLV\% }}}}$$

$$\hat{P} = \frac{1}{{1 + {\text{exp}}\left( { - \mathop \sum \nolimits_{i = 0}^{p} \beta_{i} X_{i} } \right)}}$$

For clinical application, the formulas could be made into a mobile phone or computer applet. By inputting the baseline CT_FLV/TLV%_, the prediction of the 1-year, 3-year and 5-year death probability of the patient would be obtained immediately.

These formulas were validated in the validation cohort with high accuracy. As shown in the Table 3, 15 of the 16 patients with 5-year death probability > 50% was actually died, and 13 of the 15 patients with 5-year death probability ≤ 50% was actually alive. The sensitivity, specificity and accuracy were 88.2%, 92.8% and 90.3%. Similar results were observed for 1-year death probability and 3-year death probability.

**Table 3:** Consistency of predicted 1-year, 3-year and 5-year death probability and true survival status

Consistency of predicted 5-year death probability and true survival status		
	Predicted death	Predicted alive
True death	15	2
True alive	1	13

Consistency of predicted 1-year death probability and true survival status		
	Predicted Death	Predicted Alive
True death	9	2
True Alive	0	20

Consistency of predicted 3-year death probability and true survival status		
	Predicted Death	Predicted Alive
True death	10	3
True alive	2	16

### Correlation of dynamic changes in the CT _FLV/TLV%_ and survival

**Fig. 5:**
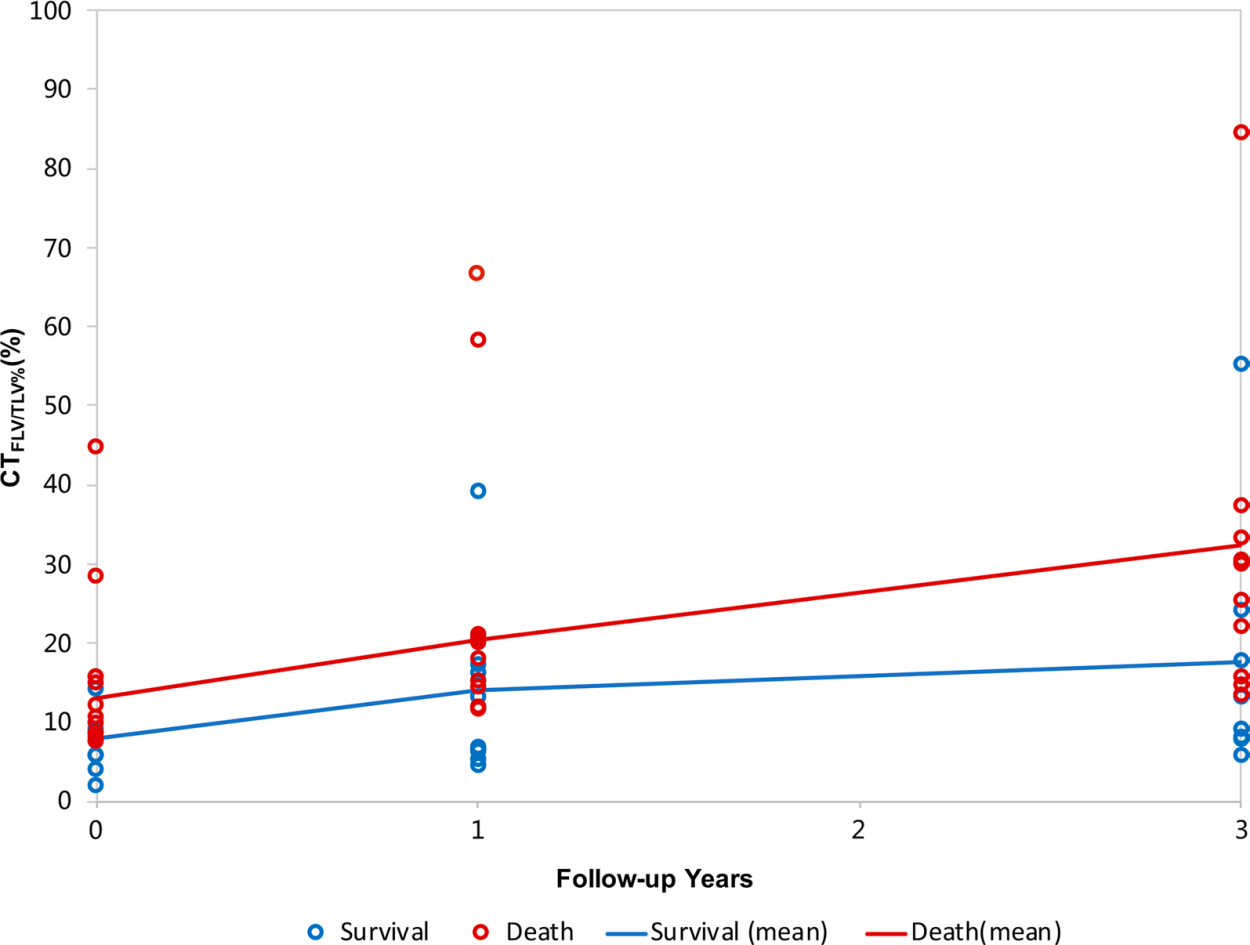
Dynamic CT_FLV/TLV%_ within 3 years. The CT_FLV/TLV%_ of individual patients as well as the mean CT_FLV/TLV%_ at baseline and 1 year and 3 years after enrollment are shown in the graph. The patients were divided into two groups according to their 5-year survival status at the end of the follow-up. The two lines are far apart from each other, indicating that the baseline CT_FLV/TLV%_ correlated with survival (P = 0.0531). The CT_FLV/TLV%_ increases with time (P = 0.0005). The two lines are nearly parallel, and there is no interaction for the CT_FLV/TLV%_ within 3 years (P = 0.1641)

In the derivation cohort, dynamic CT _FLV/TLV%_ data were available for 21 patients. At the end of the follow-up, 9 survived, and 12 died. The CT_FLV/TLV%_ at baseline and at 1 year and 3 years after diagnosis between the two groups (survival and death) were compared (Table 4 and Fig. 5). The CT_FLV/TLV%_ increased with time in both groups (P = 0.0005), and the value of the CT_FLV/TLV%_ was higher in the death group (P = 0.0531), indicating that the baseline CT_FLV/TLV%_ correlated with survival. Although only a small number of patients had dynamic HRCT images, the slopes of the two lines were almost parallel (P = 0.1641), which indicates that fibrotic lesions of the lung develop, regardless of the patients’ group. The results are consistent with the theory that once IPF is diagnosed, the progression of the disease is irreversible.

**Table 4:** Dynamic CT_FLV/TLV%_ and 5-year survival status

	Survival	Death
Patient number	9	12
Baseline CT_FLV//TLV%_(%)	7.85 ± 1.86	13.05 ± 1.66
1-year CT_FLV/FLV%_(%)	13.94 ± 4.51	20.42 ± 4.03
3-year CT_FLV/TLV%_(%)	17.69 ± 6.51	32.38 ± 5.83

## Discussion

The general prognosis of IPF is poor but varies widely among individuals. Accurate and precise prognosis prediction of IPF will guide the clinical strategy, such as the best time to start drug therapy, lung transplantation, or palliative care.

Physiological variants of the PFT, especially the FVC%pred and DLCO%pred, have been reported as predictors for the prognosis of IPF in past studies [1, 15–19]. Therefore, we identified the FVC%pred and DLCO%pred as candidate variants in our study to validate their prediction value in IPF prognosis. In our cohort, the baseline DLCO%pred was correlated with survival according to the univariate analysis, which was consistent with the results of past studies. However, the predictive value of the baseline FVC%pred failed to be validated. Although many studies have considered the FVC%pred as a predictor for prognosis [15–17], few studies have reported that the baseline FVC%pred has no dependent correlation with survival [18, 20]. Instead, the decline rate of FVC is a commonly validated predictor [17, 21, 22]. A decline in the FVC ≥ 10% is associated with an increased risk of death [17]. DLCO changes over time are also valuable in prognosis prediction and have been shown to be a better predictor of mortality than FVC changes [23]. In our study, the baseline FVC%pred showed no significant difference between the survival and death groups, but the decline rate of FVC between the groups might be different, which would influence the prognosis. However, limited by the retrospective nature of the study, we did not obtain follow-up PFT reports from the patients. Another possible reason might be the bias due to incomplete PFT data since 10 patients lost their initial PFT reports and 9 patients could not complete the PFT process. KCO, which is often written as DLCO/alveolar volume (VA), is an index of the efficiency of alveolar transfer of carbon monoxide. DLCO is often decreased in interstitial lung diseases because of diffuse alveolar capillary damage. VA is decreased due to loss of aerated alveoli. Therefore, the extent of KCO reduction is often less than that of DLCO [24]. Few studies have identified KCO as a prognostic predictor. Corte et al. reported that a decline in KCO in six months predicted early mortality in patients with idiopathic interstitial pneumonia [25]. In our study, we found that the baseline KCO%pred along with the DLCO%pred were correlated with survival. However, we did not further compare the prediction value of the two variants.

The GAP model is the most widely validated clinical prediction model for IPF. It incorporates age, sex, FVC, and DLCO into a simple point-score index and staging system, predicting 1-, 2-, and 3-year mortality [3]. The correlation between the baseline GAP and survival was confirmed in our study.

Compared with the PFT, CT is a promising examination for prognosis prediction for IPF, with the advantages of convenience and objectivity. Lynch et al. confirmed the correlation between DLCO and HRCT findings and suggested that the extent of reticulation and honeycombing on HRCT images is an important independent predictor of mortality in patients with IPF [10]. Ley et al. used the CT fibrosis score to replace DLCO and constructed a modified GAP model [8]. Salisbury et al. reported that postbaseline changes in ground glass-reticular densities correlated with changes in the FVC [9]. These findings support CT as an alternative when the PFT is unavailable or unreliable.

Although the CT images were objective, the repeatability and homogeneity of the visual assessment of CT were limited by the experience of the radiologists, and it was difficult to quantify the lesion volume precisely by artificial assessment [8, 12, 26]. Jacob et al. [13] reported that computer-derived CT variants are superior predictors of mortality than any visually scored CT parameter in patients with IPF. Therefore, computer-assisted CT evaluation is a future assessment direction, which could provide more precise and comprehensive evaluation.

Several studies have reported computer-assisted segmentation, quantification, and characterization of pulmonary fibrosis. Maldonado et al. [11] explored the application of the computer-aided lung informatics for pathology evaluation and rating (CALIPER) software to quantify parenchymal lung abnormalities on HRCT images and found that short-term volumetric longitudinal changes in serial HRCT images correlated with IPF mortality in a retrospective cohort of patients with IPF. Salisbury et al. [9] used adaptive multiple features method (AMFM) lung texture analysis software to recognize and quantify the volume of lung occupied by ground glass, ground glass-reticular, honeycombing, emphysema, and normal lung in HRCT images of patients with IPF and found that a greater volume occupied by AMFM-measured fibrosis was independently associated with an increased hazard of disease progression. A recent study compared different software programs [27] and found that the shape model-based segmentation software tool was superior to the threshold-based tool since the density of (severe) fibrosis was similar to that of the surrounding soft tissues. These studies have proven the value of computer software in quantitative analysis of CT in IPF and have revealed the close correlation of quantified severity in CT with disease severity, progression and prognosis of IPF, showed advantage of volumetric analysis of total lung as compared with visual scoring based on a few CT images. But these studies had not provided detailed prognosis prediction for an individual patient.

We used Synapse 3D, a software to quantify the volume of fibrotic lung and total lung and obtain the ratio of FLV/TLV, defined as the CT_FLV/TLV%_. We aimed to verify the correlation between the CT_FLV/TLV%_ and physiological variants and the prognosis of IPF in a retrospective cohort from our hospital and tried to provide a precise and detailed formula for the prediction of prognosis for a certain patient. The univariate Cox proportional hazards model analysis showed that the baseline CT_FLV/TLV%_ as well as physiological variants, including DLCO%pred, KCO%pred and GAP index, correlated with the survival time, but the multivariate analysis showed that the CT_FLV/TLV%_ was the only factor correlated with survival. The cutoff point at 11% CT_FLV/TLV%_ was chosen from the ROC curve, and there was a significant difference in the survival time between the CT_FLV/TLV%_ ≤ 11% and > 11% groups. Formulas to predict the probability of death at 1 year, 3 years and 5 years since the diagnosis of IPF were calculated through logistic regression based on the baseline CT_FLV/TLV%_. These formulas were validated as with high sensitivity and specificity by a validation cohort, and might provide precise predictions of prognosis for individual patients and provide a reference for clinical strategies. Patients firstly diagnosed as IPF would be evaluated by their baseline HRCT. We could get the baseline CT_FLV/TLV%_ by using the computer software, then calculate the 1-year, 3-year and 5-year death probability by using the formulas. For those patients with high risk of death, we could give them more active interventions, starting anti-fibrotic drugs as early as possible, and applying for lung transplantation.

There are still some limitations to our study. Firstly, this study was retrospective, which may have limited the sample number and data quality. The treatments given to the patients were not identical, which may also influence the assessments of prognosis. Secondly, although the formulas were confirmed to be with high sensitivity and specificity in the validation cohort from our hospital, a larger external validation cohort from multiple centers to validate the suitability of the formulas would be look forward. Thirdly, the assistance of experienced radiologists and respiratory physicians in the quantification of fibrosis or total lung volume from CT images was still required because of the lack of fully automated artificial intelligence computer software for the analysis. With the development of machine learning and artificial intelligence [28, 29], these problems might be resolved in the near future. Fourthly, since dynamic CT _FLV/TLV%_ data were available in only 32.8% (n = 21) of the total cohort (n = 64), the analysis of the correlation of the CT_FLV/TLV%_ changes with disease progression and prognosis failed to obtain a certain result. A larger sample number is needed for future research.

In conclusion, we quantified the volume of fibrotic lungs and total lungs through Synapse 3D and obtained the CT_FLV/TLV%_, analyzed factors that might influence the prognosis of patients with IPF in a retrospective cohort, and confirmed that the baseline CT _FLV/TLV%_ was significantly correlated with the survival time. Formulas to predict the probability of one to five years death of IPF patients were calculated and might serve as a reference in clinical decision-making for individual patients.

## Data Availability

The raw data supporting the conclusion of this article will be made available by the authors, without undue reservation.

## Abbreviations

IPF: Idiopathic pulmonary fibrosis
GAP: Gender-age-physiology
HRCT: High-resolution computed tomography
FVC: Forced vital capacity
FVC%pred: FVC percent predicted
DLCO: Diffusing capacity of carbon monoxide
DLCO%pred: DLCO percent predicted
KCO: Carbon monoxide transfer coefficient
KCO%pred: KCO percent predicted
FLV: Fibrotic lung volume
TLV: Total lung volume
ROC: Receiver operating characteristic
CT_FLV/TLV%_: FLV/TLV ratio
ATS: American Thoracic Society
ERS: European Respiratory Society
UIP: Usual interstitial pneumonia
6MWT: Six-minute walk test
CPI: Composite Physiologic Index
ROSE: Risk Stratification Score
PFT: Pulmonary Function Test
CADx: Computer-aided diagnosis
AUC: Area under the curve
CALIPER: Computer-aided lung informatics for pathology evaluation and rating
AMFM: Adaptive multiple features method
